# A novel engineered VEGF blocker with an excellent pharmacokinetic profile and robust anti-tumor activity

**DOI:** 10.1186/s12885-015-1140-1

**Published:** 2015-03-25

**Authors:** Lily Liu, Haijia Yu, Xin Huang, Hongzhi Tan, Song Li, Yan Luo, Li Zhang, Sumei Jiang, Huifeng Jia, Yao Xiong, Ruliang Zhang, Yi Huang, Charles C Chu, Wenzhi Tian

**Affiliations:** 1 Department of Cell Biology, Huabo Biopharm Co Ltd., Shanghai, 201203 China; 2 Department of Antibody Technology, Huabo Biopharm Co Ltd., Shanghai, 201203 China; 3 Department of Protein Science, Huabo Biopharm Co Ltd., Shanghai, 201203 China; 4 Department of Project Management, Huabo Biopharm Co Ltd., Shanghai, 201203 China; 5 The Feinstein Institute for Medical Research, North Shore-LIJ Health System, Manhasset, NY 11030 USA; 6 Department of Medicine, Hofstra North Shore-LIJ School of Medicine, Hempstead, NY 11549 USA; 7 Department of Molecular Medicine, Hofstra North Shore-LIJ School of Medicine, Hempstead, NY 11549 USA

**Keywords:** VEGF inhibitor, VEGFR1, Recombinant Fc-fusion protein, Anti-tumor therapy, Angiogenesis

## Abstract

**Background:**

Relatively poor penetration and retention in tumor tissue has been documented for large molecule drugs including therapeutic antibodies and recombinant immunoglobulin constant region (Fc)-fusion proteins due to their large size, positive charge, and strong target binding affinity. Therefore, when designing a large molecular drug candidate, smaller size, neutral charge, and optimal affinity should be considered.

**Methods:**

We engineered a recombinant protein by molecular engineering the second domain of VEGFR1 and a few flanking residues fused with the Fc fragment of human IgG1, which we named HB-002.1. This recombinant protein was extensively characterized both *in vitro* and *in vivo* for its target-binding and target-blocking activities, pharmacokinetic profile, angiogenesis inhibition activity, and anti-tumor therapeutic efficacy.

**Results:**

HB-002.1 has a molecular weight of ~80 kDa, isoelectric point of ~6.7, and an optimal target binding affinity of <1 nM. The pharmacokinetic profile was excellent with a half-life of 5 days, maximal concentration of 20.27 μg/ml, and area under the curve of 81.46 μg · days/ml. When tested in a transgenic zebrafish embryonic angiogenesis model, dramatic inhibition in angiogenesis was exhibited by a markedly reduced number of subintestinal vessels. When tested for anti-tumor efficacy, HB-002.1 was confirmed in two xenograft tumor models (A549 and Colo-205) to have a robust tumor killing activity, showing a percentage of inhibition over 90% at the dose of 20 mg/kg. Most promisingly, HB-002.1 showed a superior therapeutic efficacy compared to bevacizumab in the A549 xenograft model (tumor inhibition: 84.7% for HB-002.1 versus 67.6% for bevacizumab, P < 0.0001).

**Conclusions:**

HB-002.1 is a strong angiogenesis inhibitor that has the potential to be a novel promising drug for angiogenesis-related diseases such as tumor neoplasms and age-related macular degeneration.

## Background

Targeted tumor therapy is the focus of recent intense drug development by the pharmaceutical industry with the primary interests centered on antibody drugs [1]. However antibody and/or recombinant protein drugs with molecular weights (MWs) of over 100 kDa usually have relatively poor tumor penetration and retention capacity for which the molecular size, charge, as well as target binding affinity play important roles [2]. There are several barriers to large molecule transport in solid tumors due to disordered vasculature, tissue structure, as well as extracellular matrix (ECM). These factors, which impact penetration and retention of large molecule drugs, have to be considered when designing new molecular constructs.

Angiogenesis, the process by which the existing vascular network expands to form new blood vessels, is mainly mediated by vascular endothelial growth factor (VEGF), which upon binding with VEGF receptor (VEGFR), can induce phosphorylation of the receptors expressed in the blood vessel endothelial cells [1], thus leading to proliferation of the endothelial cells and the development of the vascular system. Under pathological conditions, VEGF-A and other members of the VEGF family including placental growth factor (PlGF) are upregulated [3-6]. Among the factors contributing to angiogenesis, VEGF-A is the main ligand driving angiogenesis, making it an important target for drug development.

Several drugs targeting VEGF have been approved for use in the treatment of cancer [7] as well as for wet age-related macular degeneration (AMD) [8]. Bevacizumab is a humanized antibody targeting VEGF-A and was approved under the trade name of Avastin in 2004 for the treatment of metastatic colon cancer [9-11] as well as several other solid tumors including lung cancers [12,13], glioblastoma [14,15], renal cancers [16], and ovarian cancers [17-19]. The main mechanism by which bevacizumab exerts anti-tumor activity is by preventing VEGF-A from binding with its receptors, thus resulting in inhibition of new blood vessel growth in tumor tissues. Bevacizumab is a humanized IgG1 with over 90% of human and less than 10% of murine components [20]. The recommended dose for bevacizumab is 5 mg/kg every 2 weeks, even though it could be detected in serum for 12 weeks [21]. Bevacizumab is the first VEGF blocker proven to improve survival by 30% in patients with metastatic colorectal cancer [22]. However due to target limitation (only targeting VEGF-A) as well as relatively poor tissue penetration because of its large size, the overall impact of bevacizumab in prolonging survival was very limited [22,23], with 5-year survival generally between 5% and 8% [23], suggesting that VEGF-A blockade alone may not be good enough to completely prevent tumor angiogenesis and corresponding tumor growth.

Aflibercept (originally called VEGF-Trap) was approved in August of 2012 under the trade name of Zaltrap for the treatment of metastatic colon cancer, and the same molecule was approved in November of 2011 under the trade name of Eylea for the treatment of AMD. Aflibercept is a recombinant fusion protein consisting of the second immunoglobulin (Ig) domain of VEGFR1 and the third Ig domain of VEGFR2, fused to the immunoglobulin constant region (Fc) portion of human IgG1 [24]. Unlike bevacizumab, aflibercept exhibits affinity for all isoforms of VEGF and PlGF [25] and exerts robust antivascular effects by rapid regression of existing tumor vessels [26], normalization of surviving mature vessels [27], and inhibition of new tumor vessel growth [28]. The anti-tumor efficacy of aflibercept has been confirmed in several solid tumor models, all demonstrating effective tumor inhibition [29]. Aflibercept has a MW of 110 kDa and has a half-life in plasma of 4-5 days [24]. The clinical benefits for aflibercept treatment of metastatic colon cancer patients are similar to bevacizumab [30].

It has been documented that the VEGF-binding affinity of VEGFR1 is 10 fold higher than that of VEGFR2 [31] and the second Ig domain of VEGFR1 is critical for VEGF binding [32]. We reasoned that a recombinant protein composed of only the second domain (D2) of VEGFR1 might retain sufficient VEGF binding, but also have better bioavailability and penetration properties due to its smaller size as compared to the previously described current generation of drugs that block VEGF. We therefore designed an expression vector that expressed a recombinant protein consisting of the D2 portion of VEGFR1 fused with the Fc portion of human IgG1. This protein was extensively characterized for its target-binding affinity, angiogenesis inhibition, and pharmacokinetic (PK) profile, as well as for its anti-tumor efficacy in several xenograft tumor models.

## Methods

### Engineering of recombinant proteins

HB-002.1 is a recombinant protein consisting of two components: one is the D2 domain of human VEGFR1 (Flt1) (P134-T226) plus 5 (S129-R133) and 2 (N227, T228) amino acids of upstream and downstream flanking sequence respectively, and the second is the Fc fragment of human IgG1. To construct the HB-002.1 expression vector, 57 nucleotides encoding the signal peptide of mouse IgG1 heavy chain were added to the 5' end of VEGFR1-D2, a Kozak sequence was added to the 5' end of the signal peptide sequence, and cloning sites, *Hin*dIII and *Eco*RI, were added to the 5' and 3' ends of the resulting sequence, respectively. This designed D2 expression cassette sequence was synthesized (GenScript) and subcloned into the *Hin*dIII and *Eco*RI sites of the pHB-Fc vector (Generay, ID: X9913T).

The recombinant Flt1[2]-Fc protein contains the VEGFR1-D2 domain (P134-T226) without the addition of flanking region amino acids, plus the Fc fragment of human IgG1.

All recombinant proteins were expressed and purified from Chinese hamster ovary (CHO) cells (Cat# CCL-61, ATCC). 5 μg of each protein were loaded on 10% SDS-PAGE gels under reducing as well as non-reducing conditions. Gels were stained with 0.3% Coomassie Brilliant Blue R-250 and destained with 20% methanol.

### Western blotting and digestion of proteins with N-glycosidase F

To validate the identity of the purified protein, Western blotting analysis was performed [33]. Briefly, different amounts of the purified protein (1, 0.5, 0.25 μg) were separated by electrophoresis in 4-12% Bis-Tris protein gels, and then transferred to a polyvinylidene difluoride membrane. The membrane was probed using antibodies specific either for Fc fragment (horseradish peroxidase (HRP)-conjugated rabbit F(ab’)2 anti-human IgG, Fc-fragment specific (ImmunoResearch Lab) or HRP*Polyclonal Rabbit Anti-Human IgG (Fc) (Cat#C030222, Cellway-Lab, Luoyang, China)), or for human VEGFR1 (Cat# 10136-RP02, Sino Biological Inc) followed by incubation with secondary antibody (HRP-conjugated Affinipure F(ab')2 Fragment Goat Anti Rabbit IgG1, F(ab')2 Fragment Specific (ImmunoResearch Lab)). Specific bands were visualized via the ECL kit according to the manufacturer’s instructions (Amersham).

To analyze the impact of glycosylation on protein activity, HB-002.1 protein (Lot#20130521, 3.62 mg/ml) diluted to 0.5 mg/ml in 100 mM of ammonium bicarbonate was incubated with N-glycosidase F (Cat#11365193001, Sigma) (5 Unit/10 μg protein) at 37°C for 18 hours. Digested and non-digested proteins were analyzed in 12% SDS-PAGE under reducing and non-reducing conditions. In parallel, the digested protein was also assayed for target binding activity, which was compared to that of the parental protein.

### Target-binding assay

Target binding affinity of HB-002.1 was measured by ELISA in Falcon 96-Well ELISA Micro Plates coated overnight at room temperature with VEGF ligands or PIGF (R&D Systems) in PBS (100 ng per well). Coated plates were blocked with 3% dry fat milk in PBS-T buffer (PBS containing 0.05% Tween-20) and then 100 μl of serially diluted HB-002.1 or bevacizumab (Lot#:N3526, Roche) or hIgG-Fc (Cat#:10702-HNAH, Sino Biological Inc) (from 5 nM to 0.0024 nM) were transferred into the plates. After incubation at room temperature for 1 hour, plates were washed 5 times with PBS-T solution, and then incubated with HRP-conjugated Fc-specific antibody (Cat#C030222, Cellway-Lab, Luoyang, China) at room temperature for 1 hour. Plates were washed 5 times with PBS-T buffer and then developed with 100 μl of HRP-substrate solution for up to 5 minutes. The reaction was stopped with 1 N H_2_SO_4,_ and the absorbance at 450 nM was determined in a standard plate reader.

To determine the kinetic target binding affinity of HB-002.1, varying amounts of VEGF-A were mixed with 0.5 nM of HB-002.1, Flt1[2]-Fc, hIgG-Fc or bevacizumab and then incubated for 2 hours at room temperature. The mixtures were transferred to VEGF-A coated plates and incubated for 1 hour at room temperature, the non-bound proteins in solution were washed away, and the amounts of HB-002.1, Flt1[2]-Fc, hIgG-Fc or bevacizumab bound to the plates were measured by HRP-conjugated rabbit anti-human IgG-Fc antibody. The kinetic binding affinities were analyzed according to the amounts of free VEGF blocker in the mixtures.

### VEGFR2 phosphorylation assay

4 ml of human umbilical vein endothelial cells (HUVECs) (Cat#HUVEC-004, ALLCELLS) in complete HUVEC-adapted medium (Cat#H-004, ALLCELLS) were incubated in 6 cm dishes at 37°C, 5% CO_2_ for 24 hours, cells were starved for 2 hours and then challenged for 15 minutes with either medium alone, or VEGF-A (20 ng/ml) only, or VEGF-A pre-incubated with varying amounts of HB-002.1. Cells were washed twice with cold PBS and then dissolved in 200 μl of lysis buffer (50 mM Tris, pH 7.4, 1% sodium deoxycholate, 1% Triton X-100, 0.1% SDS, 1 mM EDTA, pH 8.0, 150 mM NaCl). After centrifugation and quantitation, equal amounts of supernatant from each sample were subjected to Western blotting analysis using antibodies specific either for total VEGFR2 (Cat# 2479, Cell Signaling Technology) or for VEGFR2 phosphotyrosine (Cat# 3770S, Cell Signaling Technology).

### VEGF-induced HUVEC proliferation and tube formation assay

HUVEC proliferation in response to VEGF-A and the impact of HB-002.1 on cell proliferation was measured using CCK-8 kits (Cat# CK04-11, DOJINDO Laboratories) following the manufacturer's instructions. Briefly, 2000 HUVECs per well were plated in a 96-well plate, which was incubated at 37°C for 2 hours. 100 μl of reagent solution containing 20 ng/ml of VEGF-A and varying amounts of HB-002.1, bevacizumab or hIgG-Fc were transferred to the plate. Cells were cultured for 72 hours at 37°C, and then CCK-8 was added to these cultures, which were incubated for 4 additional hours followed by spectrophotometric analysis at 450 nm.

The VEGF-induced tube formation assay was conducted as previously described [34]. Briefly, 50 μl of HUVECs at 3 × 10^5^/ml in culture medium were mixed with 50 μl of culture medium containing 20 ng/ml of VEGF-A plus 1000 nM HB-002.1 protein, bevacizumab or control human IgG. The mixtures were added to 96-well plates containing 50 μl of solidified Matrigel. Plates were incubated in a cell culture incubator at 37°C for 24 hours. Tube formation was observed using an inverted phase contrast microscope (Eclipse TS100, Nikon). Images were captured with a CCD color camera (KP-D20AU, Hitachi) attached to the microscope using 40x magnification plus 1.5x amplification by the CCD camera. The tube length in three different fields was measured using Image-Pro Plus software (Version 6.0, Media Cybernetics).

### Angiogenesis analysis

The impact of HB-002.1 on angiogenesis was investigated using a transgenic zebrafish embryonic angiogenesis model [35]. Briefly, the tested protein or control drugs were microinjected into the common cardinal vein of zebrafish at 48 hours post-fertilization (hpf). The subintestinal vessels (SIVs) were visualized under a Multi-Purpose Zoom Microscope (Nikon AZ100), and the area of the SIVs at 72 hpf was measured as mean fluoresence intensity (MFI) using NIS-Elements D imaging software. The percentage of angiogenesis inhibition was calculated as (MFI of vehicle treated SIVs - MFI of drug treated SIVs)/MFI of vehicle treated SIVs x 100.

### Pharmacokinetic analysis

16 BALB/c mice (female, age of 4-5 weeks, body weight of 18-20 g) received a subcutaneous (s.c.) injection of 50 μg HB-002.1 protein (~2.5 mg/kg mouse) and bled at 1, 2, 4, 6, 24, 48, 72, and 144 hours after injection. Levels of HB-002.1 in the plasma were measured by ELISA assay using human VEGF165 (R&D Systems) as capture protein and HRP-anti-human Fc (Jackson ImmunoResearch Lab) as the detection antibody.

### *In vivo* efficacy study

Mouse xenograft tumor models using human Colo-205 and A549 cancer cells were applied to the investigation of the *in vivo* efficacy of HB-002.1. Cells purchased from ATCC were resuspended in serum-free medium. BALB/c nude mice were ordered from Shanghai SLAC Laboratory Animal Co. Ltd. The animals were specific pathogen free and approximately 4 - 5 weeks old upon arrival at PharmaLegacy Laboratories. The procedures that were applied to animals in this protocol had been approved by PharmaLegacy Laboratories IACUC before the execution of the study. Approximately 5 × 10^6^ cells in 200 μl of serum-free medium/matrigel (50:50 v/v) were injected s.c. in the right flank of each of the 70 mice for each model under anesthesia by 3 - 4% isoflurane. When the average tumor volume reached 100 - 200 mm^3^, 50 mice bearing tumors of suitable size were randomized into 5 groups (10 mice per group) according to tumor volume and body weight. Mice were treated with two different doses (5 mg/kg, 20 mg/kg) of HB-002.1 or control drugs by intraperitoneal (i.p.) injections twice weekly for four weeks except for doxorubicin which was given only in one injection. Tumor volume and body weight were measured twice a week until the termination of the study. Tumor growth inhibition (TGI%) = (1-(change in mean treated tumor volume/change in mean control untreated tumor volume)) × 100. Tumor weight measured at time of mice sacrifice.

### Histology analysis

Tumors were harvested and sectioned at the end of the experiments. Tumor sections were subsequently dewaxed and rehydrated. After quenching endogenous peroxidase activity, sections were immunohistochemically stained with respective antibody. Stained sections were dehydrated in alcohol and xylene, and then mounted. The procedure for hematoxylin and eosin (H&E) staining of tumor sections was as follows: dewaxing in xylene, gradient ethanol dehydration, hematoxylin staining, rinsing with tap water, counterstaining with eosin, rinsing with ethanol, gradient ethanol dehydration, and vitrification with xylene. Immunohistochemical staining was performed using antibodies specific for CD31 (Cat#: ab9498, Abcam) followed by goat anti-mouse secondary antibody (Cat#: KIT5002, Fuzhou Maixim) and goat anti-rabbit secondary antibody (Cat#: KIT5005, Fuzhou Maixim), respectively. The microvessel density was quantified by the visual approximation technique, which involved manual counting vessels in three different microscope fields at 10x magnification. The histology results were analyzed by a pathologist on a single-blind basis. For tumor necrosis evaluation on H&E stained slides, homogenous staining in pink or pale color without cellular profiles/outline were considered necrotic cells, while cellular profiles/outlines with dark blue nuclei were considered healthy cells.

### Statistics

Statistical software used for data analysis and presentation was SAS 9.3 (SAS Institute), Prism 5 (GraphPad Software), and Excel 11 (Microsoft). Binding curves were calculated and presented using Prism 5 nonlinear regression least squares fit sigmoidal dose-response variable slope (also known as four-parameter dose-response) curves. Comparisons between different treatment groups in HUVEC proliferation was performed using a two-way analysis of variance (ANOVA), which included the main effects of treatment group and log10 concentration, as well as the treatment group x log10 concentration interaction. Upon finding a significant interaction effect, separate one-way ANOVA comparisons were carried out at each concentration. If a significant difference was found, then Tukey’s multiple comparisons were used. Comparisons between different treatment groups in tube formation by one-way ANOVA provided a F-test with a small P value (P = 0.0015) supporting subsequent Tukey’s multiple comparison test. Comparisons between control (vehicle-treated) and different treatment groups for inhibition of zebrafish angiogenesis were made by Dunnett’s multiple comparison test. *In vivo* tumor volumes and weights were expressed as mean ± standard error of the mean or geometric mean with 95% confidence interval. Comparisons between different *in vivo* treatments and control PBS treated mice for changes in tumor weights were made by Mann-Whitney two-tailed test. For tumor volume, repeated measures (RM) ANOVA with a mixed models approach was used to determine if the treatment groups behaved differently across time (i.e. the “group x time” interaction). A log_10_ transformation of tumor volume was used to satisfy the required underlying assumptions of this statistical model. Since graphical analysis and theoretical considerations suggest that tumor volume grows logarithmically, such that its rate of growth decreases over time, a log_10_transformation was applied to day (specifically, log_10_of Day +1), and included as a linear main effect, as well as in the interaction term with group. The model contained one repeated “within subjects” factor of time, a “between animals” factor of treatment group, and the group x time interaction. Both group and time were considered fixed effects in each of the RM ANOVA models, as necessarily was, the group x time interaction. Upon finding a significant difference, interest only focused on the comparison of the treatment groups to control (PBS), but not amongst each other. To calculate the statistical significance of treatments on TGI%, we calculated the ratio of tumor volume at Day 35 relative to Day 0 for each mouse, followed by a log transformation of this ratio to achieve normality (log Day35/Day0), which is analytically equivalent to looking at percent change in tumor volume, but is more suited to conventional analysis. ANOVA was then used to compare the mean log ratios with the Student-Newman-Keuls test to make multiple comparisons. P < 0.05 was considered significant. For CD31 staining of tumor sections, only group descriptive statistics were calculated. No inferential statistical comparisons were performed since the sample size was so small (n = 3).

## Results

### Engineering and production of HB-002.1

**Figure 1:**
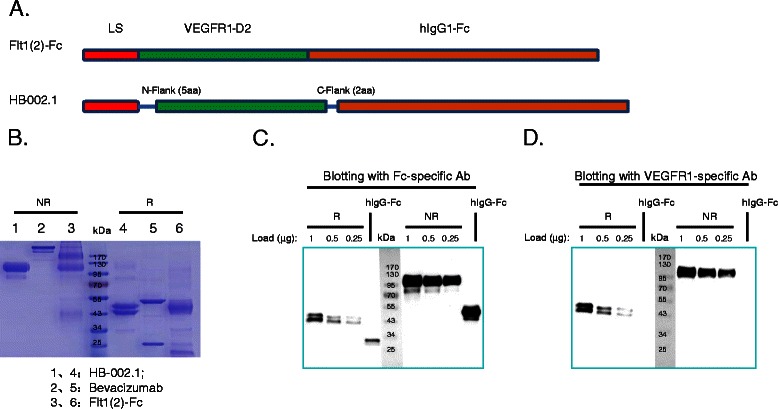
**Engineering and production of HB-002.1. (A)** Diagram of HB-002.1 engineered structure. Illustration on top represents Flt1[2]-Fc consisting of the D2 domain only fused with the Fc portion of human IgG1. HB-002.1 contains the D2 domain plus 5 and 2 amino acids at the 5' and 3' flanking region respectively. Signal peptide derived from the heavy chain of mouse IgG1 (LS) was included for both constructs. **(B)** SDS-PAGE gel analysis. Three proteins were included in the analysis: HB-002.1 (Lane 1, 4); Bevacizumab (Lane 2, 5); Flt1[2]-Fc (Lane 3, 6). 5 **μ**g of each protein were loaded under reducing and non-reducing conditions. **(C-D)**, Western blot analysis. 0.25, 0.5, and 1 **μ**g of HB-002.1 protein and 1 **μ**g of hIgG-Fc were resolved on 10% SDS-PAGE gels under reducing (R) or non-reducing (NR) conditions, transferred to a polyvinylidene difluoride membrane, and probed with Fc-specific **(C)** or VEGFR1-specific **(D)** antibody (Ab). For comparison, protein MW size markers are shown in kDa.

It has been documented that the second Ig-like domain (D2) of human VEGFR1 (Flt1[2]) is critical to VEGF binding [32], however the purified Flt1[2] fused with Fc did not bind to VEGF at all, and neither did truncated protein containing the first 2 domains (Flt1[1,2]) or that containing domains 2 and 3 (Flt1[2,3]) [32]. Only protein containing domains 1-3 had full VEGF binding activity comparable to that of the whole extracellular portion of wild type VEGFR1. This phenomenon was confirmed as well by Barleon et al [36], revealing the requirement of VEGF binding for the first three Ig-like domains. Based on these studies, we designed the HB-002.1 protein in which 5 flanking amino acids (S129-R133) at the N-terminal and 2 amino acids (N227, T228) at the C-terminal of the D2 domain were included with D2 (Figure 1A). The D2 domain-only (Flt1[2]-Fc) was also expressed as a control for VEGF binding assay.

The HB-002.1 and Flt1[2]-Fc proteins were produced in CHO cells upon transfection with the corresponding construct. The secreted proteins were purified and resolved in 10% SDS-PAGE gels showing MWs of HB-002.1 and Flt1[2]-Fc at ~110 kDa in non-reducing conditions, and ~45 kDa in reducing conditions (Figure 1B), both relatively larger than the calculated MW, which is most likely due to glycosylation since there are two N-linked glycosylation sites in the D2 domain. Bevacizumab resolved in the correct MW positions (Figure 1B).

To confirm the identity of the proteins, Western blotting was performed using antibodies specific for Fc (Figure 1C) or VEGFR1 (Figure 1D), showing specific bands for each specified portion of the protein at different protein loading amounts.

### HB-002.1 has strong binding affinity to VEGF-A

**Figure 2:**
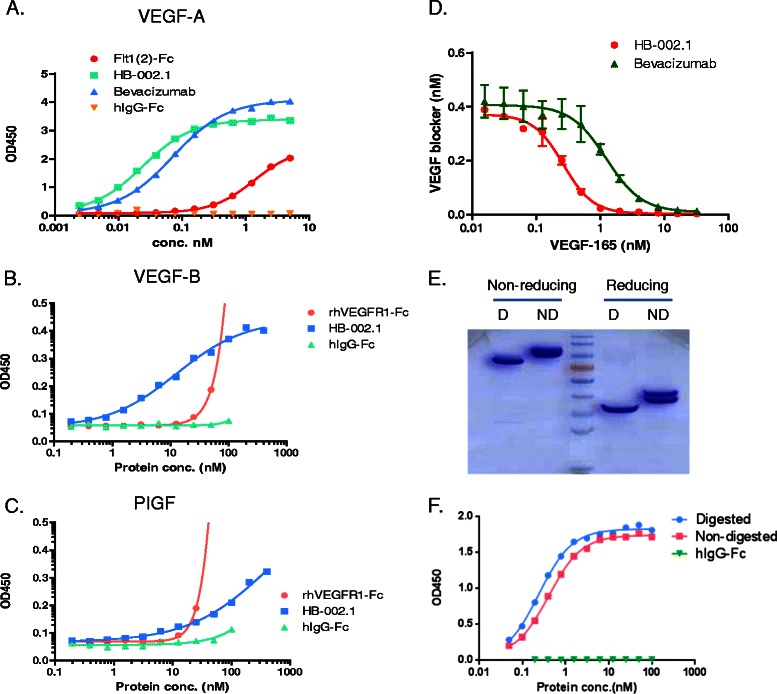
**Target binding activity of HB-002.1.** Target binding activity of intact as well as deglycosylated HB-002.1 was analyzed by ELISA. **(A)** Binding to VEGF-A was compared to bevacizumab and Flt1(2). hIgG-Fc was used as negative control. **(B-C)** Binding to VEGF-B **(B)** and PIGF **(C)** was compared with rhVEGFR1-Fc. **(D)** Kinetic binding affinity was measured by equilibrium binding assays that measures unbound HB-002.1 or bevacizumab after incubation of 0.5 nM of HB-002.1 or bevacizumab with varying amounts of VEGF-165. **(E)** HB-002.1 deglycosylated by treatment with N-glycosidase F **(D)** or not deglycosylated (ND) was separated by SDS-PAGE under reducing or non-reducing conditions and visualized by staining with Coomassie Brilliant Blue. **(F)** VEGF-A binding affinity was compared between intact (non-digested) and deglycosylated (digested) HB-002.1.

HB-002.1 was first analyzed for its binding affinity to VEGF-A and compared with that of Flt1[2]-Fc and bevacizumab. The data showed that HB-002.1 had a high affinity with a half maximal effective concentration (EC50) of 24 pM, which was 3-fold higher than that of bevacizumab (EC50 = 72 pM) (Figure 2A). As expected, Flt1[2]-Fc only had a minimal binding activity to VEGF-A, confirming a binding requirement for the flanking sequence. Binding activity of HB-002.1 to VEGF-B and PIGF was also investigated by ELISA, showing a modest binding to VEGF-B (Figure 2B) but low binding to PIGF (Figure 2C).

To determine the target-binding kinetics of HB-002.1, equilibrium binding assays were performed in which varying amounts of VEGF-A were mixed with 0.5 nM of HB-002.1 or bevacizumab, and the unbound HB-002.1 or bevacizumab was measured by ELISA using VEGF-A coated plate, revealing that HB-002.1 displays an equilibrium dissociation constant (K_D_) of 180 pM, whereas bevacizumab has a K_D_ of 890 pM (Figure 2D).

Since two different-sized bands were observed both in SDS-PAGE gels and in Western blots, we wondered if this was due to N-linked glycosylation and if this glycosylation might have an impact on VEGF binding. To address these questions, HB-002.1 protein was digested with N-glycosidase F and then resolved in 10% SDS-PAGE gels, which showed a single band under reducing conditions and a smaller size single band under non-reducing conditions when compared to that of non-digested parental protein (Figure 2E). This confirmed our hypothesis that the doublet bands were due to N-linked glycosylation. The digested protein retained similar VEGF-binding activity to that of parental protein (Figure 2F), indicating glycosylation is not essential for high affinity binding, which is consistent with the report by Barleon et al [36].

### HB-002.1 dose-dependently inhibited VEGF-induced VEGFR2 phosphorylation, HUVEC proliferation and tube formation

**Figure 3:**
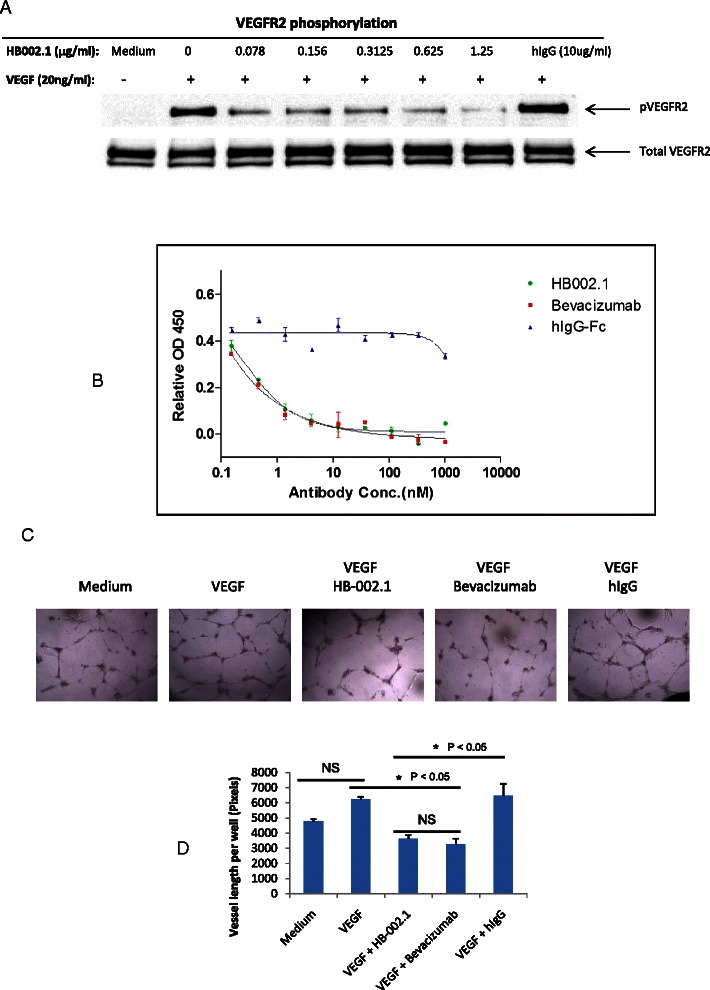
***In vitro*biological activity. (A)** HB-002.1 inhibited VEGF-induced VEGFR2 phosphorylation as revealed with immunoblotting assay. This experiment was repeated three times, all showing a similar pattern of inhibition in VEGFR2 phosphorylation. **(B)** Inhibition of VEGF-induced HUVEC cell proliferation was analyzed with the CCK-8 kit, a colormetric assay. Assay was repeated three times with duplicate wells for each concentration. Representative assay is shown. Significant differences between HB-002.1 and hIgG-Fc (P < 0.05 at all except lowest dose), and bevacizumab and hIgG-Fc (P < 0.05 at all doses) were observed. Bevacizumab and hIgG-Fc was used as positive and negative controls, respectively. **(C)** Representative microscopic images of HUVEC tube formation in Matrigel are shown for medium alone, or for medium plus VEGF with or without HB-002.1, Bevacizumab, or hIgG. **(D)** Tube formation was quantified by counting the total vessel length per field. Data were collected from duplicate wells (mean ± standard deviation). Statistical significance was evaluated by ANOVA and Tukey’s multiple comparison test. Differences between Medium versus VEGF and VEGF + HB-002.1 versus VEGF + Bevacizumab were not significant (NS). Differences between VEGF + HB-002.1 or VEGF + Bevacizumab versus Medium or VEGF + hIgG were significantly different (P < 0.05).

Due to the strong VEGF binding affinity, we anticipated that HB-002.1 must also have strong blocking activity against VEGF-induced VEGFR2 phosphorylation as well as the resulting cell proliferation and tube formation. As shown in Figure 3A, while strong phosphorylation was observed with VEGF addition and VEGF plus hIgG, the induced phosphorylation was sequentially diminished following addition of sequentially increasing amounts of HB-002.1, which is comparable to that of bevacizumab showing a dose-dependent inhibition of VEGFR2 phosphorylation (Tyr951/1175) in HUVECs [37].

Comparisons between different treatment groups in HUVEC proliferation (Figure 3B) by two-way analysis of variance (ANOVA) provided a F-test with a small P value (P < 0.0004) supporting subsequent evaluation for differences among treatment groups. Significant inhibition, as compared to hIgG-Fc, in VEGF-induced HUVEC proliferation was observed in a dose-dependent manner for HB-002.1 (P < 0.05 at all except lowest dose), which was comparable to that of bevacizumab (P < 0.05 at all doses) (Figure 3B). The same phenomenon was also observed for VEGF-induced tube formation (Figure 3C, D), for which HB-002.1 had a significant and comparable inhibition to that of bevacizumab (P < 0.05) as compared to hIgG, suggesting a strong blocking activity of HB-002.1 in VEGF-mediated cell biological activity.

### HB-002.1 dose-dependently inhibited *in vivo* angiogenesis

**Figure 4:**
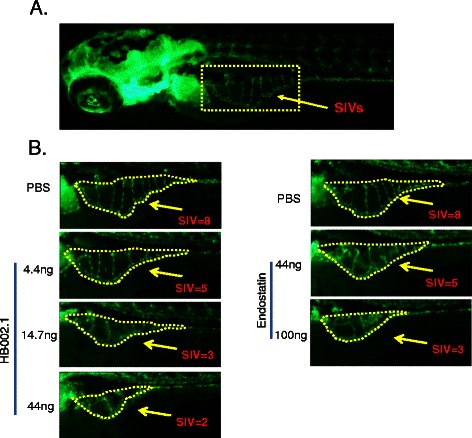
***In vivo*angiogenesis inhibition. (A)** Subintestinal vessels (SIVs) under normal conditions are shown. **(B)** HB-002.1 at different doses (4.4, 14.7, 44 ng) (left) was injected into the blood flow during embryogenic development of zebrafish. Endostatin at two doses (44, 100 ng) was included in the study as positive controls (right). Representative Images from one of the ten zebra fishes in each group are shown. Arrows point to the number of SIVs in each group.

Using a transgenic zebrafish embryonic angiogenesis model [35], the impact of HB-002.1 on *in vivo* angiogenesis was investigated and showed a dramatic reduction in number of SIVs. While 7-8 SIVs were usually observed in zebrafish at 72 hpf (Figure 4A), a decreased number of SIVs was observed when treated with HB-002.1 (Figure 4B). The level of inhibition versus vehicle group reached 7.5 (±3.5) % (P > 0.05), 15.2 (±3.3) % (P < 0.01), and 21.4 (±2.4) % (P < 0.001) for HB-002.1 at the doses of 4.4, 14.7, 44 ng, respectively. Because bevacizumab specifically binds human VEGF and its activity against zebrafish VEGF is not known, a broad spectrum angiogenesis inhibitor, endostatin, known to inhibit angiogenesis in this model [38], was used as a positive control showing a 9.7 (±2.8) % and 20.1 (±2.6) % inhibition at the dose of 44 and 100 ng respectively (Figure 4B). These results suggest a strong angiogenesis inhibition activity for HB-002.1.

### HB-002.1 has an excellent pharmacokinetic profile

HB-002.1 has a much smaller molecular mass than current VEGF inhibitors, bevacizumab and aflibercept (~80 vs. ~160 and ~110 kDa, respectively), thus it might have a shorter half-life and worse PK profile compared to these drugs. To address these questions, 2.5 mg/kg of HB-002.1 were injected s.c. into mice (n = 16) and plasma taken at different time points post-injection were analyzed for HB-002.1 levels by ELISA. The results indicated that HB-002.1 has a half-life of 5 days (Table 1), similar to that of therapeutic antibodies, such as bevacizumab (~6 days after 9.3 mg/kg s.c. injection) [39], and Fc-fusion proteins [24]. Furthermore, HB-002.1 has additional excellent PK properties, with a maximal concentration (*C*_*max*_) of 20.27 μg/ml, mean residence time (MRT) of 7.5 days, and total area under the curve concentration (AUC) of 81.46 μg · days/ml (Table 1). This is comparable to that observed for therapeutic antibody, bevacizumab, starting at a higher dose (9.3 mg/kg), with a C_max_ of 74.1 μg/ml, MRT of 8.74 days, and an AUC of 682 μg · days/ml [39,40]. Interestingly, HB-002.1 PK properties are better than that published for therapeutic fusion protein, despite aflibercept starting at a higher dose (4 mg/kg), with a C_max_ of 16 μg/ml and an AUC of 36.28 μg · days/ml (Table 2) [24]. Considering the lower isoelectric point (pI = 6.7) of HB-002.1 compared to pI = 7.6 and 8.82 for bevacizumab [37] and aflibercept [24], respectively (Table 2) and smaller MW of HB-002.1, the excellent PK properties may be due to better tissue penetration of the protein.

**Table 1:** **Pharmacokinetic parameters of HB-002.1**

AUC (g · days/ml)	MRT (hr)	T_1/2_(hr)	C_max_(μg/ml)
81.46	180	120	20.27

Note: AUC, area under the curve concentration; MRT, mean.

residence time; T_1/2_, half-life; C_max_: maximal concentration.

**Table 2:** **Comparison of selected PK parameters among VEGF inhibitors**

Inhibitor	pI	Dose (mg/kg)	C_max_(μg/ml)	AUC (μg · days/ml)	Reference
HB-002.1	6.7	2.5	20.3	81.46	This study
Bevacizumab	7.6	9.3	74.1	682	39
Parental VEGF-Trap	9.4	4.0	0.05	0.04	24
VEGF-TRAP_∆B1_	9.1	4.0	1.3	1.36	24
VEGF-TRAP_∆B2_	8.9	4.0	2.65	5.42	24
Aflibercept	8.82	4.0	16	36.28	24

### HB-002.1 exhibited robust *in vivo* anti-tumor activity

**Figure 5:**
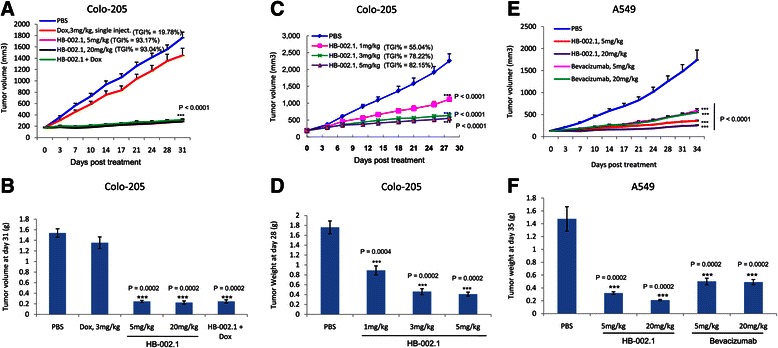
***In vivo*efficacy study. (A-B)** HB-002.1 treatment even at low dose (5 mg/kg) reached maximal inhibition of tumor growth in Colo-205 s.c. xenograft model (n = 10 for each group) **(A)** P < 0.0001 versus PBS treatment for tumor volume over time. **(B)** P = 0.0002 versus PBS for tumor weight at time of sacrifice. Treatment was started when tumor volume reached to 150-200 mm^3^, twice a week, through i.p. injection. **(C-D)** In Colo-205 xenograft model (n = 10 for each group), varying amounts of HB-002.1 (1, 3, 5 mg/kg) were tested on effect on tumor growth. Significant tumor growth inhibition was observed in the lowest dose (1 mg/kg, P < 0.0001 for tumor volume and P = 0.0004 for tumor weight versus PBS). **(E-F)** Therapeutic efficacy of HB-002.1 in A549 xenograft model (n = 10 for each group) was analyzed and compared with that of bevacizumab at doses of 5, 20 mg/kg. HB-002.1 at low dose (5 mg/kg) exhibited superior efficacy than bevacizumab at high dose (20 mg/kg) (TGI% = 78.02 for HB-002.1, versus 67.55 for bevacizumab) (Table 3).

The anti-tumor activity of HB-002.1 was evaluated in two different tumor models, Colo-205 and A549, representing human colorectal cancer and lung cancer respectively. BALB/c nude mice bearing these xenograft tumors were treated with HB-002.1 as well as control drugs by i.p. injection, twice a week, for up to four weeks. Tumor volume was measured twice a week and compared between groups. In the Colo-205 xenograft model, HB-002.1 was compared to doxorubicin, a potent tumor chemotherapeutic drug that has widespread use clinically and has demonstrated efficacy in several human tumor xenograft models [41-43]. Compared to the PBS vehicle group, treatment with the positive control drug, doxorubicin, at 3 mg/kg by single bolus i.p. injection slightly inhibited the tumor growth (TGI% = 19.78) (Figure 5A), while treatment with HB-002.1 at 5 or 20 mg/kg i.p. twice weekly showed a significant tumor growth inhibition, as indicated by the decrease in tumor volume (TGI% = 93.17 at 5 mg/kg, TGI% = 93.04 at 20 mg/kg, P < 0.0001) (Figure 5A) and tumor weight (P = 0.0002) (Figure 5B). Interestingly, the combination therapy of HB-002.1 with doxorubicin did not show any synergistic increase in efficacy, being equivalent to HB-002.1 treatment alone (Figure 5A-B). Thus, promisingly, even at the low dose (5 mg/kg), HB-002.1 treatment alone still reached maximal inhibitory effect in this model, whereas bevacizumab was reported to only reach TGI% = 55 at the dose of 4.0 mg/kg [44], suggesting a robust anti-tumor activity for HB002.1.

To determine an effective dosing regimen of HB-002.1, three doses were applied to the Colo-205 model, which in comparison to PBS, showed a TGI% on day 28 of 55, 78.2, 82.1 at doses of 1.0, 3.0, and 5.0 mg/kg, respectively (Figure 5C, P < 0.0001). This was better than that reported for bevacizumab with 33, 41, and 44 TGI% at doses of 1.2, 2.5, and 4.0 mg/kg, respectively, in the same model [44]. Additionally, the TGI% for aflibercept at a much higher dose of 25 mg/kg was reported to be only 62-75 [41]. Tumor weight at the end of the study also showed a dramatic and dose-dependent decrease in the HB-002.1 treated group (Figure 5D, 1.0 (P = 0.0004), 3.0 and 5.0 (P = 0.0002) mg/kg dose). These studies clearly revealed that HB-002.1 has remarkable anti-tumor activity, suggesting that HB-002.1 may be a potential alternative therapy for colorectal cancer.

The anti-tumor activity of HB-002.1 was also evaluated in the A549 xenograft model, and compared in parallel to that of bevacizumab, for two different dosages (5, 20 mg/kg). While bevacizumab showed a similar but dramatic inhibitory effect at both doses, similar to that previously reported [45], the inhibition was more pronounced for HB-002.1 even at the low dose (5 mg/kg) (Figure 4E-F). When compared to that treated with PBS, the TGI% was 78.02 and 84.71 for HB-002.1 at 5 and 20 mg/kg, respectively, which was significantly better than bevacizumab at the same doses, 64.46 and 67.55 TGI%, respectively (Table 3).

**Table 3:** **Tumor growth inhibition in A549 xenograft model**

Drugs	Mean Tumor Volume	Growth inhibition (TGI%)	P value vs PBS	P value vs Bevacizumab
HB-002.1, 5 mg/kg	2.72 (2.51-2.91)	78.02	<0.0001	<0.0001
HB-002.1, 20 mg/kg	1.89 (1.66-2.16)	84.71	<0.0001	<0.0001
Bevacizumab, 5 mg/kg	4.40 (3.59-5.40)	64.46	<0.0001	NA
Bevacizumab, 20 mg/kg	4.02 (3.36-4.81)	67.55	<0.0001	NA
PBS	12.38 (9.87-15.54)	0.00	NA	NA

Sample size = 10 for each group.

Mean Tumor Volume = geometric mean with 95% confidence interval in parentheses. Geometric mean is shown because statistical analyses utilized log-transformed data that best modeled tumor volume growth.

P value was calculated as described in Methods.

P value vs Bevacizumab is compared to same dose of HB-002.1.

NA = not applicable.

### HB-002.1 induced tumor growth inhibition is associated with decreased microvessel density and increased necrosis of tumor cells

**Figure 6:**
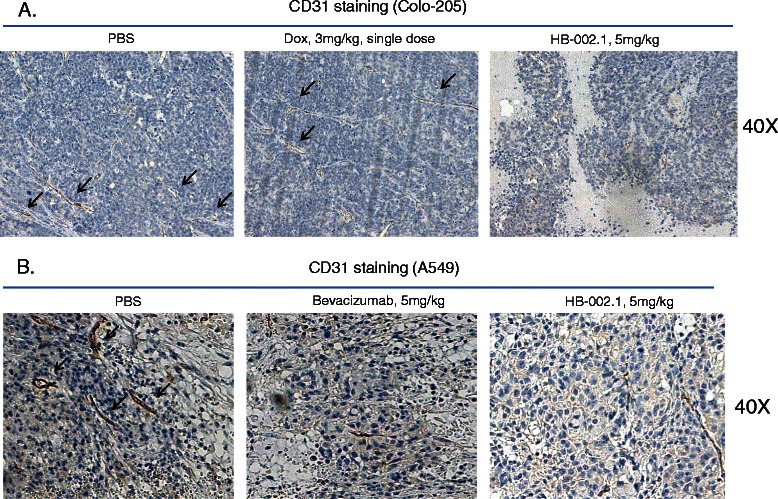
**Inhibition of tumor angiogenesis.** Tumor angiogenesis analysis was performed by CD31 staining. Significant reduction in new blood vessel formation (black arrows) was observed in HB-002.1 treated Colo-205 **(A)** as well as A549 **(B)** tumors, respectively. Representative fields of CD31 staining in tumors treated with PBS (left) or doxorubicin (upper middle) or HB-002.1 (right) are shown at 40x magnification (40X).

To determine whether the inhibitory effect of HB-002.1 on tumor growth was associated with angiogenesis inhibition in tumor tissues as a result of VEGF blockade, microvessel density was analyzed by staining tumor tissue sections with CD31-specific antibody. Treatments with 5 mg/kg of HB-002.1 inhibited formation of CD31^+^ microvessels when compared to that of the vehicle group in the Colo-205 or A549 xenograft models (Figure 6, Table 4). This inhibition was quantified by measuring the percentage of positive CD31 staining area against the total tumor area by the visual approximation technique (1.9% for HB-002.1 vs. 6.2% for PBS in the Colo-205 model; 0.7% for HB-002.1 vs. 7.1% for PBS in the A549 model) (Table 4). Promisingly, HB-002.1 showed a more potent effect on CD31^+^ vessel formation than that for doxorubicin in the Colo-205 model and that for bevacizumab in the A549 model (Figure 6, Table 4). This inhibition of microvessel formation in these models is similar to that reported by others for bevacizumab and aflibercept [41,44,45].

**Table 4:** **Percentage of positive CD31 staining in tumor section**

Group	% of CD31 in Colo-205	% of CD31 in A549
PBS	6.2 (+/- 2.1)	7.1 (+/-3.2)
Doxorubicin, 3 mg/kg	6.8 (+/- 3.6)	not done
HB-002.1, 5 mg/kg	1.9 (+/- 0.9)	0.7 (+/- 0.2)
Bevacizumab, 20 mg/kg	not done	3.9 (+/- 2.9)

Note: Average (+/- SEM) from 3 samples in each group is shown.

**Figure 7:**
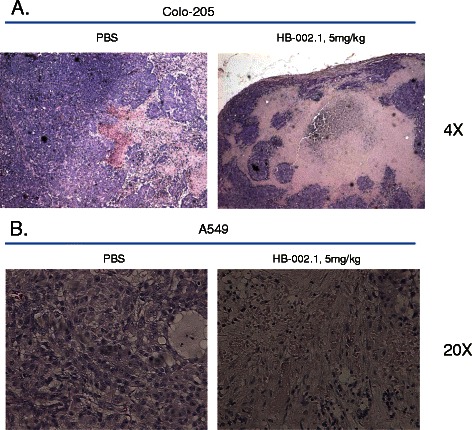
**H&E staining analysis.** H&E staining for HB-002.1 treated tumor sections at low dose (5 mg/kg) was presented and compared to that of PBS treated group. Large regions of tumor necrosis were observed in Colo-205 **(A)** as well as A549 **(B)** tumor sections respectively. Representative fields of H&E staining are shown at 4x **(A)** and 20x **(B)** magnification for Colo-205 and A549 tumor sections, respectively.

To confirm that tumor cell necrosis resulted because of a decreased nutritional supply, H&E staining analysis was conducted on tumors removed at the end of the studies. As shown in Figure 7A-B, while little tumor necrosis was observed in vehicle-treated tumors, large regions of necrosis, as exhibited by decreased or absent hematoxylin-stained (blue) tumor cell nuclei and disorganized cell outlines, were observed in HB-002.1-treated tumors. This is comparable to that described for bevacizumab [45] and aflibercept [41].

## Discussion and conclusion

In the current study, we engineered a new smaller-sized recombinant VEGF-inhibiting Fc-fusion protein, HB-002.1 (Figure 1), which had an excellent VEGF-binding activity (Figure 2) and PK profile (Tables 1 and 2) comparable or better than the current generation of VEGF-inhibiting drugs. This translated into excellent *in vivo* anti-tumor efficacy, as shown by its superior therapeutic efficacy as compared to bevacizumab in the A549 xenograft model (Figure 5E-F, Table 3). Even at low dose (5 mg/kg), the inhibition mediated by HB-002.1 was still two-fold better than that of bevacizumab at high dose (20 mg/kg), although the better efficacy could be partially contributed by HB-002.1 cross-reaction with mouse VEGF, which does not occur with bevacizumab. More promisingly, HB-002.1 still reached 50% inhibition of tumor growth in the Colo-205 model at a dose as low as 1 mg/kg, suggesting a robust anti-tumor activity for HB-002.1 (Figure 5C).

Achieving effective concentrations within solid tumor masses has been challenging for large molecule drugs [46]. Better penetration and longer retention in the targeted area of the body are ideal parameters for large molecule drugs to reach optimal therapeutic efficacy. It has been known that the impact factors on penetration, retention, as well as other PK properties, include molecular size, charge, valence, and target binding affinity. For a given protein drug, the rate of diffusion through tumors is inversely correlated to the molecular weight [47,48]. scFv fragments diffuse approximately 6 times faster than IgG due to their smaller size. However proteins with molecular mass less than 60 kDa, which is the threshold for glomerular filtration, will be subject to quick renal clearance resulting in shorter half-lives.

Molecular charge affects tumor distribution substantially. The defined pI range for optimal tumor penetration is between 5 and 9 [49]; out of this range, therapeutic proteins are prone to immobilization by electrostatic interactions with the vascular endothelium and/or ECM [49]. Tumors have disordered tissues with regard to vasculature, interstitial fluid pressure, cell density, tissue structure and composition, and ECM components [50]. Tumor ECM is richer in collagen and stiffer than normal tissue ECM [51]. Proteins with high positive charge will be easily adhered to highly negatively charged proteoglycans found in the ECM. For example, the parental VEGF-Trap molecule before aflibercept had a high pI (9.4) and poor PK properties, with a *C*_max_ of only 0.05 μg/ml and total AUC of 0.04 μg × days/ml (Table 2) [24]. Realizing the effectiveness of parental VEGF-Trap may be affected by its high pI, basic amino acids were removed to create VEGF-Trap_∆B1_, followed by removal of additional basic amino acids to create VEGF-Trap_∆B2_, and finally replacing an entire protein domain with a less basic protein domain to create aflibercept (Table 2) [24]. This molecular engineering caused a steady reduction of the pI from 9.4 to 8.82 and resulted in a concomitant improvement in the PK profile, with a final C_max_ of 16 μg/ml and an AUC of 36.28 μg × days/ml (Table 2) [24]. Thus, for a given therapeutic protein, the ideal pI should be near physiologic pH as molecules with neutral charge diffuse more readily.

To our knowledge, the robust anti-tumor efficacy of HB-002.1 seen in xenograft models and superior efficacy compared to bevacizumab could be attributed to three reasons. The first reason is the molecular weight which is only ~80 kDa, much smaller than both aflibercept (~110 kDa) and bevacizumab (~160 kDa). The relatively small size makes it easier to penetrate into tumor tissues and accumulate, resulting in better bioavailability. The second reason is the pI of HB-002.1 is near neutral, which is the most ideal pI for a recombinant protein as inverse correlation between the pI and the PK profile has been seen with aflibercept (Table 4) [24]. The third reason might be due to cross reactivity of HB-002.1 with mouse VEGF, which is not seen with bevacizumab.

In conclusion, we have designed a novel recombinant VEGF blocker designated as HB-002.1, which has an excellent PK profile and robust anti-tumor activity as compared to the current generation of VEGF blockers. The accumulated research data exhibited in this report warrant further preclinical analysis of HB-002.1, which will set the basis for clinical investigation.

## Abbreviations

AMD: Age-related macular degeneration
ANOVA: Analysis of variance
AUC: Area under the curve concentration
CHO: Chinese hamster ovary
C_max_: Maximal concentration
D2: Second domain
EC50: Half maximal effective concentration
ECM: Extracellular matrix
Fc: Immunoglobulin constant region
H&E: Hematoxylin and eosin
hpf: Hours post-fertilization
HRP: Horseradish peroxidase
HUVECs: Human umbilical vein endothelial cells
Ig: Immunoglobulin
i.p.: Intraperitoneal
K_D_: Equilibrium dissociation constant
MRT: Mean residence time
MW: Molecular weight
pI: Isoelectric point
PK: Pharmacokinetic
PlGF: Placental growth factor
RM: Repeated measures
s.c.: Subcutaneous
SIV: Subintestinal vessel
TGI%: Tumor growth inhibition
VEGF: Vascular endothelial growth factor
VEGFR: VEGF receptor
